# SPIX: A new software package to reveal chemical reactions at trace amounts in very complex mixtures from high‐resolution mass spectra dataset

**DOI:** 10.1002/rcm.9015

**Published:** 2021-01-20

**Authors:** Edith Nicol, Yao Xu, Zsuzsanna Varga, Said Kinani, Stéphane Bouchonnet, Marc Lavielle

**Affiliations:** ^1^ Laboratoire de Chimie Moléculaire, CNRS – IP Paris Ecole polytechnique Route de Saclay Palaiseau 91128 France; ^2^ Centre de Mathématiques Appliquées, CNRS – IP Paris Ecole polytechnique Route de Saclay Palaiseau 91128 France; ^3^ Inria École polytechnique 1 Rue Honoré d'Estienne d'Orves Palaiseau 91120 France; ^4^ Laboratoire National d'Hydraulique et Environnement (LNHE), Division Recherche et Développement Electricité de France (EDF) 6 Quai de Watier Chatou Cedex 01 78401 France

## Abstract

**Rationale:**

High‐resolution mass spectrometry based non‐targeted screening has a huge potential for applications in environmental sciences, engineering and regulation. However, it produces large datasets for which full appropriate processing is a real challenge; the development of processing software is the last building‐block to enable large‐scale use of this approach.

**Methods:**

A new software application, SPIX, has been developed to extract relevant information from high‐resolution mass spectral datasets. Dealing with intrinsic sample variability and reducing operator subjectivity, it opens up opportunities and promising prospects in many areas of analytical chemistry. SPIX is freely available at: http://spix.webpopix.org.

**Results:**

Two features of the software are presented in the field of environmental analysis. An example illustrates how SPIX reveals photodegradation reactions in wastewater by fitting kinetic models to significant changes in ion abundance over time. A second example shows the ability of SPIX to detect photoproducts at trace amounts in river water, through comparison of datasets from samples taken before and after irradiation.

**Conclusions:**

SPIX has shown its ability to reveal relevant modifications between two series of large datasets, allowing, for instance, the study of the consequences of a given event on a complex substrate. Most of all – and it is to our knowledge the only software currently available allowing this – it can reveal and monitor any kind of reaction in all types of mixture.

## 1 INTRODUCTION

High‐resolution mass spectrometry (HRMS) is now experiencing unprecedented growth. It appeared in the early 1970s with dual‐focus devices combining magnetic and electrostatic fields, and continued its development with the introduction of time‐of‐flight (TOF), Orbitrap and Fourier transform ion cyclotron resonance (FT‐ICR) analyzers. While FT‐ICR mass spectrometers remain the most accurate today, high‐end QTOFs and QEx Orbitraps provide accuracies below 3 ppm. The resolution of an analyzer reflects its ability to separate ions with close *m*/*z* values. High‐resolution analyzers can thus differentiate isobaric ions: i.e. ions with the same nominal mass but different exact masses, and therefore different chemical formulae, such as N_2_^+•^ (*m*/*z* 28.0056) and CO^+•^ (*m*/*z* 27.9944). High resolution is a very valuable asset: it not only greatly improves the selectivity and specificity of “traditional” detection and quantification methods (in comparison with low‐resolution analyzers), but also greatly facilitates structural elucidation by assigning raw formulae to the detected ions.^1^

More recent use of high resolution takes advantage of its ability to separate isobaric ions, in an attempt to break free from separation methods – mainly gas (GC) or liquid chromatography (LC), or more rarely capillary electrophoresis or ion mobility – so as to expand the range of molecules detectable in a single analysis. This is particularly interesting in the context of non‐targeted analyses – in which the operator does not know which molecules are likely to be present in a sample – because the choice of a chromatographic system focuses the analysis of certain classes of compounds based on their properties (volatile or not, polar or apolar, large or small, etc.) and thereby introduces a bias attributable to the subjectivity of the analyst. Direct introduction into the ion source without prior pretreatment or chromatographic separation was shown to be a useful alternative for rapid and comprehensive diagnosis of environmental samples, but this approach remains very challenging due to the extreme complexity of environmental matrices and the large number of contaminants likely to be present.^2^ On direct introduction of a mixture, various molecules are simultaneously ionized, resulting in mass spectra yielded by the overlapping of spectra of the detectable species. Thus, complex mixture analyses provide mass spectra that can contain tens or hundreds of thousands of ions, even with soft ionization techniques such as electrospray ionization, atmospheric pressure ionization or atmospheric pressure photoionization; these spectra are of no possible use to the operator without the help of adapted software. Finding a molecule showing significant change between two conditions (upstream/downstream or after treatment, for instance) in its trace amounts in an environmental sample is like looking for a needle in a haystack. Being able to quickly evaluate all the chemical consequences of an industrial accident on the biotope can be crucial to decision‐making. In these situations, non‐targeted HRMS‐based screening is one of the last resorts for identifying unexpected or unknown contaminants.^3^, ^4^, ^5^, ^6^, ^7^, ^8^ This approach has recently been evaluated in a comprehensive collaborative study organized by the NORMAN association, in which a total of 18 institutes from 12 European countries analyzed an extract of the same water sample collected from the Danube River. The results revealed that non‐targeted analytical techniques were already widespread and that practices were substantially harmonized between the participants, but that data processing remained complicated and time‐consuming.^9^ Among the main recommendations formulated to improve the non‐targeted approach is the development of robust user‐friendly processing software. Likewise, AQUAREF – the French national reference laboratory for aquatic environment monitoring, which works in close concert with other European reference laboratories – published guidelines for HRMS untargeted analysis, for which SPIX could be a powerful tool.^10^

The first part of this article discusses the notions of uncertainty and subjectivity related to untargeted analysis. The second part presents the general working principle of SPIX software. The third part is dedicated to the presentation of results obtained on real samples. It discusses the strengths and limitations of the software and its specificities compared with the few programs currently commercially available. A brief overview of current computational and statistical approaches to extract relevant information from the *big data* of mass spectrometry analyses is provided in SI‐1 (supporting information); it describes the approaches of Kendrick^11^, ^12^ and van Krevelen,^13^, ^14^, ^15^ as well as multivariate statistical analysis.^16^, ^17^, ^18^, ^19^, ^20^, ^21^, ^22^ Multivariate analysis tools enable global understanding of many concomitant variables and of their inter‐correlations. Metabolomics processing pipelines often include univariate and multivariate statistical approaches. Univariate analysis is usually used as a pre‐processing step, while multivariate analysis is used for classification of samples or features. For example, principal component analysis is used to characterize differences of two groups of metabolomics GC/MS data for the diagnosis of gastric cancer. The Wilcoxon rank sum test showed the marker metabolites specific to the tumor group. Multivariate analysis, specifically principal component analysis, successfully divided the two groups of samples of normal and malignant gastric tissue.^23^ A comprehensive workflow for univariate analysis of LC/HRMS data was developed to follow human adult urinary metabolome variations. Univariate analysis was used as a preprocessing step: nonparametric hypothesis testing was used to assess correlations with covariables and the Wilcoxon test was used to calculate the median differences between genders. The univariate *p*‐value results together with multivariate importance in projection evidenced that there were 108 urine metabolites whose concentrations varied with either age, body mass index or gender.^24^ Concerning direct infusion mass spectrometry, a comprehensive workflow for data processing and quality control was developed for metabolomics analysis of cardiac tissue extract. It can be used for different metabolomics analyses as it focuses on the correction of intra‐ and inter‐batch variations and offers best‐practice workflows and rigorous quality assessment. The data processing steps include the Wilcoxon test and multivariate analysis.^25^ These applications could be extended to environmental samples; however, no approach has been reported using univariate or multivariate analysis which focuses on the kinetics of compounds in HRMS datasets. The concept behind multivariate analysis is different from that of the SPIX software: the latter aims at observing all statistically relevant variables individually. Examples of SPIX applications are given below.

## 2 NOTIONS OF UNCERTAINTY AND SUBJECTIVITY IN MODERN UNTARGETED ANALYTICAL APPROACHES, AND INTRODUCTION TO SPIX

To illustrate the functionality of the SPIX software, it is necessary to address the notions of uncertainty and subjectivity that are fundamental in analytical chemistry. We propose to take an example in environmental chemistry. Consider a plant located on the bank of a river; it may be a treatment plant or, on the contrary, a source of pollution. The question is whether its presence significantly alters the composition of the water. The question seems simple enough, but providing a relevant answer is much less so. The conventional approach is to take water samples upstream and downstream of the plant, analyze them chemically and compare the results. This approach, while scientifically reasonable, nevertheless raises many questions at each step of the process. How many samples are needed to take account of the spatial and temporal variability of upstream and downstream water composition? Where, when and how to sample? What sample preparation to adopt, given that each choice of solvent, filter, solid‐phase extraction column, chromatographic protocol and mass spectrometry ionization mode conditions the results of the analysis by favoring detection of certain molecules based on their size or polarity? Every single step in the analytical process introduces metrological uncertainties related to the measuring instruments used (balances, pipettes, etc.), but also to the so‐called “matrix effect”: i.e. the matrix of the reference used to validate the method is generally not rigorously identical to the matrix being analyzed. Stochastic biases and uncertainties are also caused by adsorption, evaporation, etc. The proliferation of sources of error obliges analysts to use internal standards to reduce the overall uncertainty of the results and try to conform to industry‐specific standards. Limiting the subjectivity in a method needs to make no assumptions at all, which is in contradiction with the use of an internal standard; thus, the analytical scientist is left with choosing between limiting subjectivity or limiting uncertainties. To the problem of uncertainties must be added that of operator subjectivity, at two main levels. As mentioned above, this subjectivity comes into play before measurement: when the operator establishes the analytical protocol, choices are made, conditioned by assumptions – the operator's own or those of third parties – as to what might have contaminated the water of the river. Even if the method is not “targeted” (i.e. specifically designed for the selective detection of given analytes), it cannot be considered totally “non‐targeted” as there is no effective protocol capable of extracting and detecting everything simultaneously (e.g. both polar and apolar molecules) and any selected protocol effectively excludes some potential analytes. This will lead the analyst to try to minimize sample preparation, with the dual objective of limiting uncertainties and of reducing operator‐induced subjectivity; an immediate consequence of this simplification is to increase the complexity of the data. For example, mass spectra recorded from environmental samples will be much more complex if the sample is introduced directly into the mass spectrometer without prior purification and separation. A point that is generally much less considered is operator subjectivity in interpreting results, especially when the data are complex and voluminous, when it comes to manually integrating a peak or comparing two chromatograms or two mass spectra, for example.

In 2019, a visual trial devoted to subjectivity evaluation was carried out during a European winter school on mass spectrometry, on a panel of 37 people with a strong scientific background in analytical chemistry. It consisted of a series of one‐minute projections of two images with 5 to 22 differences; panelists were asked to note the number of differences that they were able to spot. Some images were quite simple (pictures with modified areas) while others were very complex (fractals containing very small differences within complex areas). A set of simulated mass spectra containing 15 differences (variations in peak intensity, addition and removal of peaks) was presented to the panel – in triplicate and not consecutively – without prior notice. The variability between the results of these triplicates gave an average standard deviation of 2.3 observed differences per individual, with mean and median values of 9.6 and 9.7, respectively, and a range of 0–19. Considering the variability between panelists, a standard deviation of 20.6 differences was determined over the whole dataset, with mean and median values of 85.6 and 90, respectively, for a total 148 differences to be identified. The number of observed differences ranged from 31 to 122. The number of differences identified varied to the point that one operator would conclude that two spectra were almost identical while another would consider them significantly different!^26^

The problem is substantially more complicated when comparing not only spectra but series of spectra corresponding, for example, to samples taken upstream and downstream of a treatment plant. A large variation in an ion count between upstream and downstream spectra may not be significant if the magnitude of variation is equal within and between the downstream and upstream populations; changes in the abundance of this ion reflect only the intrinsic chemical variability of the environment and are not a relevant marker of the impact of the plant. On the other hand, a slight change in the abundance of an ion between “upstream and downstream spectra” may be significant if the abundance is almost constant within each population; it then reflects a real effect of the plant on water quality. The SPIX software was created to remedy the observed fact that it is impossible for an operator to determine what makes sense based on simple observation of complex datasets, especially since the data are subject to intrinsic variability. The aim is to extract relevant information from numerous complex datasets. As explained below, the software can identify significant differences between mass spectra series and track the kinetic evolution of reagents, unknown reaction intermediates and reaction products at low concentrations in complex mixtures.

## 3 MATERIALS AND METHODS

### 3.1 The SPIX software

SPIX was developed in matlab 2018a (MathWorks, Natick, MA, USA). A stand‐alone version is freely available on the website (http://spix.webpopix.org). The source code can be made available on request. Prior to performing any statistical analysis of the data, pre‐processing is required to identify and align significant peaks in the data. The method used for detection and alignment actually depends on the type of data available:

- When the device provides data in xml format, these data have already been filtered and contain only the most significant peaks. These peaks are then aligned by using the mspalign function of the Bioinformatics Toolbox (matlab) with the “shortest‐path” option.
- When the data obtained are raw data (e.g. xy Bruker format in the present study), i.e. intensities measured on a fine and regular grid, the following algorithm is used: considering *K* series to analyze, the approximate positions of the significant peaks are first roughly determined by building a single series, consisting of the maximum intensities of the *K* series at all data points, and by thresholding this series. This procedure is used to determine disjoint segments in which the peaks of each of the *K* series are located.

The position and intensity of each of these peaks are then estimated for each spectrum by fitting a model of the form *A* exp(−*α*(*x* − *m*)) for which the maximum value *A* is reached for *x = m*.

SPIX can be used in essentially two situations. The first one allows evidencing some modifications in the composition of a complex mixture over time. The focus here is on how the abundance of certain species varies as a function of a given parameter (time, pH, reagent, etc.). The objective is twofold: to detect ions with significantly varying abundance (in terms of statistical relevance), and to describe how the abundance varies by kinetic modeling. After aligning the peaks as previously described, different kinetic models, including various patterns associated with compound degradation and formation and reaction intermediates, are fitted to the data. A library including seven typical kinetics profiles is currently available; examples of graphical representations are provided in SI‐2 (supporting information). The selected model minimizes the Bayesian information criterion. The coefficient of determination *r* ^2^ is calculated to quantify the part of the variability of the data explained by the model and an ANOVA assesses whether this part of explained variability is statistically significant. The *p* value of the *F*‐test and the *r* ^2^ value are represented graphically so as to easily visualize ions with abundance accurately fitting a kinetic model.

SPIX also permits two series of samples collected under two experimental conditions to be compared. The objective is to identify the ions with significant differences in intensity and to quantify these differences. The algorithm first consists of identifying the peaks considered significant: i.e. present and above a given threshold in at least one of the two conditions. For an ion detected in this way, the procedure is as follows. First, the series are locally shifted so that all the peaks are aligned. The maximum intensity at the peak is estimated for each spectrum by fitting a model of the form *A* exp(−*α*(*x* − *m*)) for which the maximum value *A* is reached when *x = m*. This provides two series of values that can be compared on statistical tests. A *t*‐test detects differences in the mean while a nonparametric Wilcoxon test more generally detects whether the peak intensity tends to be higher in one condition than in the other. A graphical representation of the *p* values obtained for all the peaks detected, as well as of the size effects (i.e. differences in mean values between the two conditions), provides quick visualization of the chemically significant differences and the statistical relevance of the differences.

Blank correction can be done as follows. The user chooses as a threshold, a ratio and a percentile. By default, the median of intensities is used for the calculations (*p* = 0.5). For the given percentile, the ratio is defined as:

$$r_{p}\left(m/z\right)=\frac{B_{p}\left(m/z\right)}{S_{p}\left(m/z\right)}$$

with *B*_*p*_(*m*/*z*) being the percentile of order *p* of the blank intensities' maximum and *S*_*p*_(*m*/*z*) the percentile of order *p* of the experimental data intensities' maximum. If the peak intensity is higher than *S*_*p*_(*m*/*z*) (as a threshold value) in at least one of the experiment spectra, it will be kept as a peak; if not it will be ignored.

With .mat or .xml files, SPIX occupies about 500 MB (it is the matlab runtime that takes up all the space). With .xy formats the .mat conversion stage has to be added (sequentially): if a sub‐repertory (time_0 for example) is 250 MB, then SPIX occupies about 750 MB of memory. It does not represent the total volume of all sub‐repertories because SPIX loads them and converts them one by one. In all cases, it works very well on a standard PC.

### 3.2 Chemicals, reagents, irradiation processes and analysis

**FIGURE 1:**
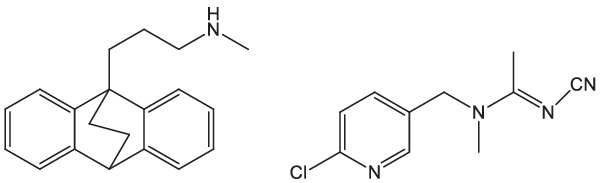
Chemical structures of maprotiline (left) and acetamiprid (right)

The ability of SPIX to extract relevant information from sets of complex high‐resolution mass spectra is illustrated in two experiments. The first concerned peroxide‐photocatalyzed degradation of maprotiline (an antidepressant drug) in a wastewater treatment pilot plant. In this case, the comparison aimed at revealing reagents, intermediates and products using kinetic models, from mass spectra recorded at different irradiation times. The second experiment concerned UV irradiation of acetamiprid (a neonicotinoid insecticide) in a complex mixture of aqueous fulvic acid to simulate river water; it aimed at revealing acetamiprid photoproducts at trace levels and evaluating the impact of UV treatment on dissolved organic matter. The comparison covers two datasets, for spectra recorded before and after irradiation. The chemical structures of maprotiline and acetamiprid are shown in Figure 1. File SI‐3 (supporting information) describes the chemicals, sampling and irradiation processes used for the two experiments.

### 3.3 HRMS analysis

An ultrahigh‐resolution mass spectrometer (FT‐ICR SolarixXR 9.4 T; Bruker Daltonics, Bremen, Germany) was used for direct infusion mass spectrometry analysis. The electrospray ionization (ESI) source was set in positive mode and solutions were injected using an automated Acquity HPLC system (Waters, Saint Quentin en Yvelines, France). The injection volume was 10 μL. Elution was carried out using a 0.002 mL/min flow of water/acetonitrile/formic acid (H_2_O/ACN/FA; 50/50/0.1). Nitrogen was used as nebulizer and as drying gas, set at 1 bar pressure and 4 L/min flow rate, respectively. The drying gas temperature was set at 180°C. The capillary voltage and endplate offset potential were set at −4500 and −500 V, respectively. Ions were accumulated for 0.2 s in the collision cell, and 50 scans were summed. The resolution was set at 4 Mpt (million data points) on a scan range from *m*/*z* 57 to *m*/*z* 1000 in order to obtain a resolution greater than 400 000 at *m*/*z* 200. A tune mix (Agilent Technologies, Les Ulis, France) was used for mass calibration. Exact formulae were assigned with error < 1 ppm.

## 4 RESULTS AND DISCUSSION

### 4.1 Highlighting chemical reactions in complex mixtures

**FIGURE 2:**
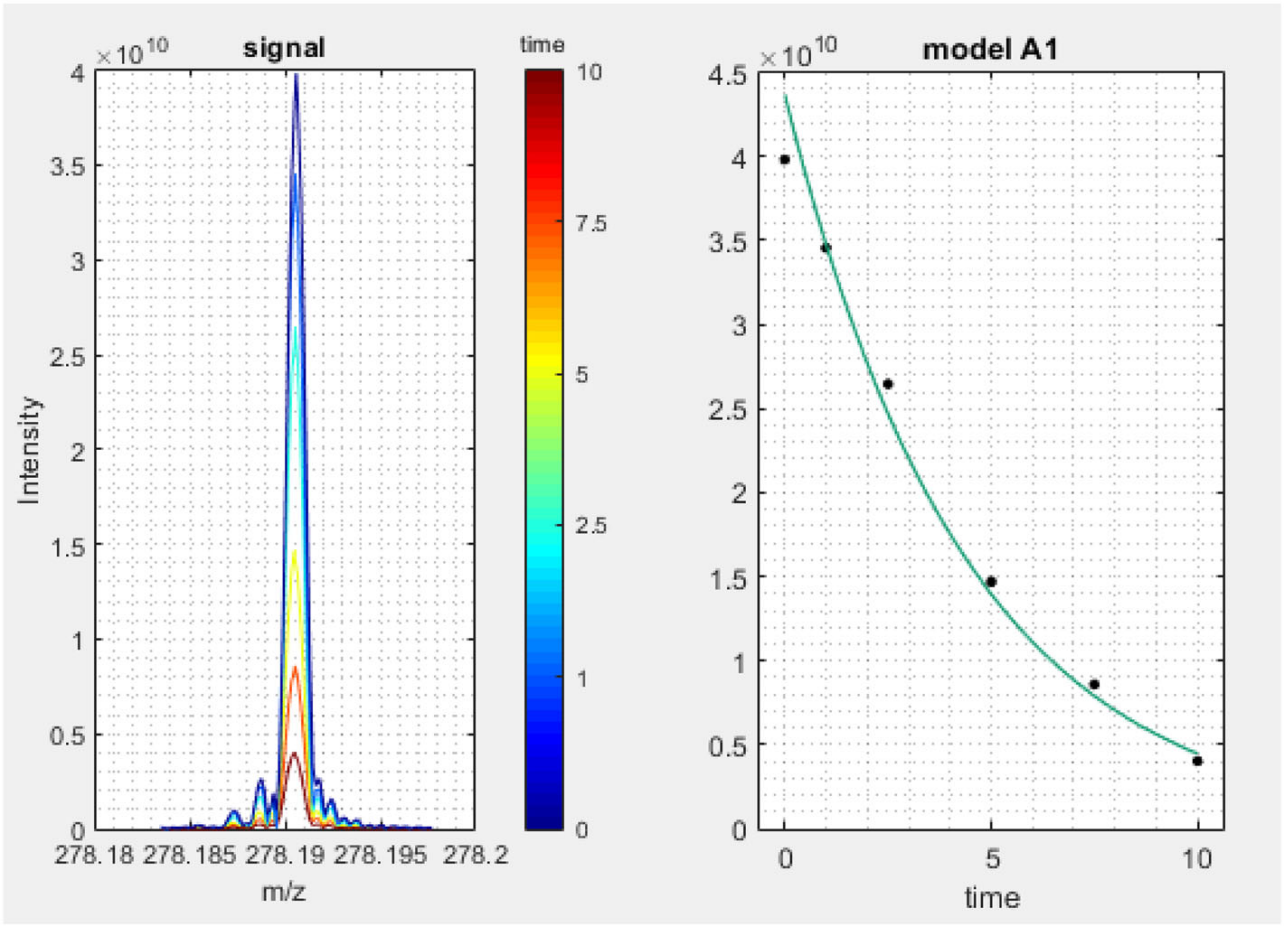
*m*/*z* 278.19056 signal (protonated maprotiline) extracted for each irradiation time and fitting model associated with degradation kinetics: 8.19 × 10^−5^ + 4.37 × 10^10^ × exp(−0.23*x*); *r* ^2^ = 0.991

Degradation of maprotiline in wastewater under an advanced oxidation process (peroxide/UV) was carried out in a pilot plant, with the aim of testing the ability of SPIX to follow the degradation of contaminants and the evolution of their transformation products. This pilot plant was set up by FACSA, a Spanish company operating water treatment plants, to design, optimize and compare novel water treatment processes; the operational parameters and analytical conditions are given in SI‐3 (supporting information). Considering that the abundances of reagents, intermediates and products of a chemical reaction are not expected to evolve stochastically, an original way to extract relevant information from untargeted analysis consists of filtering the results based on ion abundance trends. Briefly, A‐type models are selected to detect molecules with decreasing abundance during the reaction while B‐type and C‐type models detect the products and intermediates, respectively. The software detects all significant changes over time and provides a kinetic model; statistical data can be exported for further analysis in the table format described in SI‐4 (supporting information). As a first example, from one set of samples using the software default threshold (1.2 × 10^8^), SPIX automatically extracted the *m*/*z* 278.19056 signal (protonated maprotiline) for each irradiation time, and associated the fitting model referred to as A1 in SPIX with *r* ^2^ > 0.99 (Figure 2).

A first attempt to extract transformation products from the background noise, conducted on the basis of one set of measurements, gave a few maprotiline‐related peaks, but a thorough study of the raw data showed that using data from duplicate measurements yielded a kinetic model better fitting ion intensity evolution. The aim was to maximize the relevant data obtained while minimizing the number of parallel measurements, to gain valuable analysis time. Using two parallel measurements for each irradiation time, 88 ions fitting one of the SPIX kinetic models were extracted with default threshold intensity 1.2 × 10^8^; this list was reduced to 12 ions keeping *m*/*z* fitting a kinetic model with *r* ^2^ > 0.9 (Table 1), these fittings being also those corresponding to the lowest *p* values. The formulas were assigned using Bruker software based on accurate mass measurements (sub‐ppm accuracy) and isotopic pattern‐matching. All the extracted *m*/*z* values were related to maprotiline or its photoproducts (oxidized compounds); they corresponded to singly charged ions with ^12^C and ^13^C isotopic contributions. One signal (*m*/*z* 92.73055) corresponded to an artifact related to the harmonics of the *m*/*z* 278.19054 signal, resulting from signal digitization and Fourier transformation, a phenomenon previously described and explained by Mathur and O'Connor.^27^ To study the threshold effect and determine whether additional photoproducts would be found if more peaks were considered, the threshold was halved (6 × 10^7^) and the same methodology was applied. Thus 197 peaks were selected by SPIX and the data were ordered according to statistical relevance. Then 23 peaks were selected on the criterion *r* ^2^ > 0.9 (see Table 1). Here again, the *m*/*z* values were all related to maprotiline and its photoproducts; a second artifact (*m*/*z* 93.06499) was found and attributed to the harmonics of *m*/*z* 279.19386.

**TABLE 1:** Ions extracted and associated kinetic models related to the photodegradation of maprotiline in wastewater with *r* ^2^ > 0.9 (data ordered by decreasing intensity)

*m*/*z*	*r* ^2^	*p* value	Model^a^	Maprotiline‐related	Ion formula	Intensity	Relative intensity (%)
With intensity > 1.2 × 10^8^							
278.19054	0.93	5.25 × 10^−6^	A1	Yes	C_20_H_24_N^+^	8 350 669 911	100.0
279.19386	0.94	3.82 × 10^−6^	A1	Yes	C_19_H_24_N^^13^C^+^	1 643 233 281	19.7
294.18552	0.98	3.91 × 10^−7^	C1	Yes	C_20_H_24_NO^+^	1 126 960 171	13.5
276.17486	0.92	7.58 × 10^−5^	C1	Yes	C_20_H_22_N^+^	451,170,002	5.4
292.16992	0.94	2.73 × 10^−5^	B2	Yes	C_20_H_22_NO^+^	352,390,361	4.2
92.73055	0.92	1.48 × 10^−5^	A1	Yes	^b^	339,591,819	4.1
310.18038	0.94	4.09 × 10^−6^	B1	Yes	C_20_H_24_NO_2_^+^	228 987 461	2.7
295.18881	0.97	2.30 × 10^−6^	C1	Yes	C_19_H_24_NO^^13^C^+^	218 499 045	2.6
280.19727	0.94	3.44 × 10^−6^	A1	Yes	C_18_H_24_N^^13^C_2_^+^	157 067 516	1.9
308.16472	0.91	1.69 × 10^−4^	B2	Yes	C_20_H_22_NO_2_^+^	140 798 543	1.7
344.18594	0.95	1.52 × 10^−5^	B2	Yes	C_20_H_26_NO_4_^+^	135 672 030	1.6
342.17029	0.95	1.50 × 10^−5^	B2	Yes	C_20_H_24_NO_4_^+^	112 723 518	1.3
With 1.2 × 10^8^ > intensity > 6 × 10^7^							
360.18088	0.96	8.69 × 10^−6^	B2	Yes	C_20_H_26_NO_5_^+^	95 678 296	1.1
312.19604	0.94	2.71 × 10^−5^	C1	Yes	C_20_H_26_NO_2_^+^	84 376 971	1.0
328.19096	0.94	2.74 × 10^−5^	C1	Yes	C_20_H_26_NO_3_^+^	79 243 551	0.9
318.17025	0.94	2.46 × 10^−5^	B2	Yes	C_18_H_24_NO_4_^+^	75 240 290	0.9
302.1753	0.98	6.44 × 10^−7^	C1	Yes	C_18_H_24_NO_3_^+^	66 900 122	0.8
93.06499	0.92	9.63 × 10^−6^	A1	Yes	^b^	66 023 322	0.8
242.15416	0.95	1.34 × 10^−5^	B2	Yes	C_16_H_20_NO^+^	63 982 463	0.8
326.17543	0.92	8.04 × 10^−5^	C1	Yes	C_20_H_24_NO_3_^+^	50 496 456	0.6
300.17259	0.95	1.27 × 10^−6^	A1	Yes	C_20_H_23_NNa^+^	20 928 662	0.2
139.09569	0.91	9.43 × 10^−4^	C2	Yes	[C_20_H_24_N]^2+^	7 061 477	0.1
336.14927	0.91	1.76 × 10^−5^	A1	No	C_23_H_18_N_3_^+^	104 081 459	1.2

^a^ Currently available SPIX kinetic models are given in SI‐2 (supporting information).

^b^ *m*/*z* 93.06499 and *m*/*z* 92.73055 signals correspond to artifact peaks related to the harmonics of the *m*/*z* 278.19054 and *m*/*z* 279.19386 ions, respectively; they resulted from signal digitization.^27^

**FIGURE 3:**
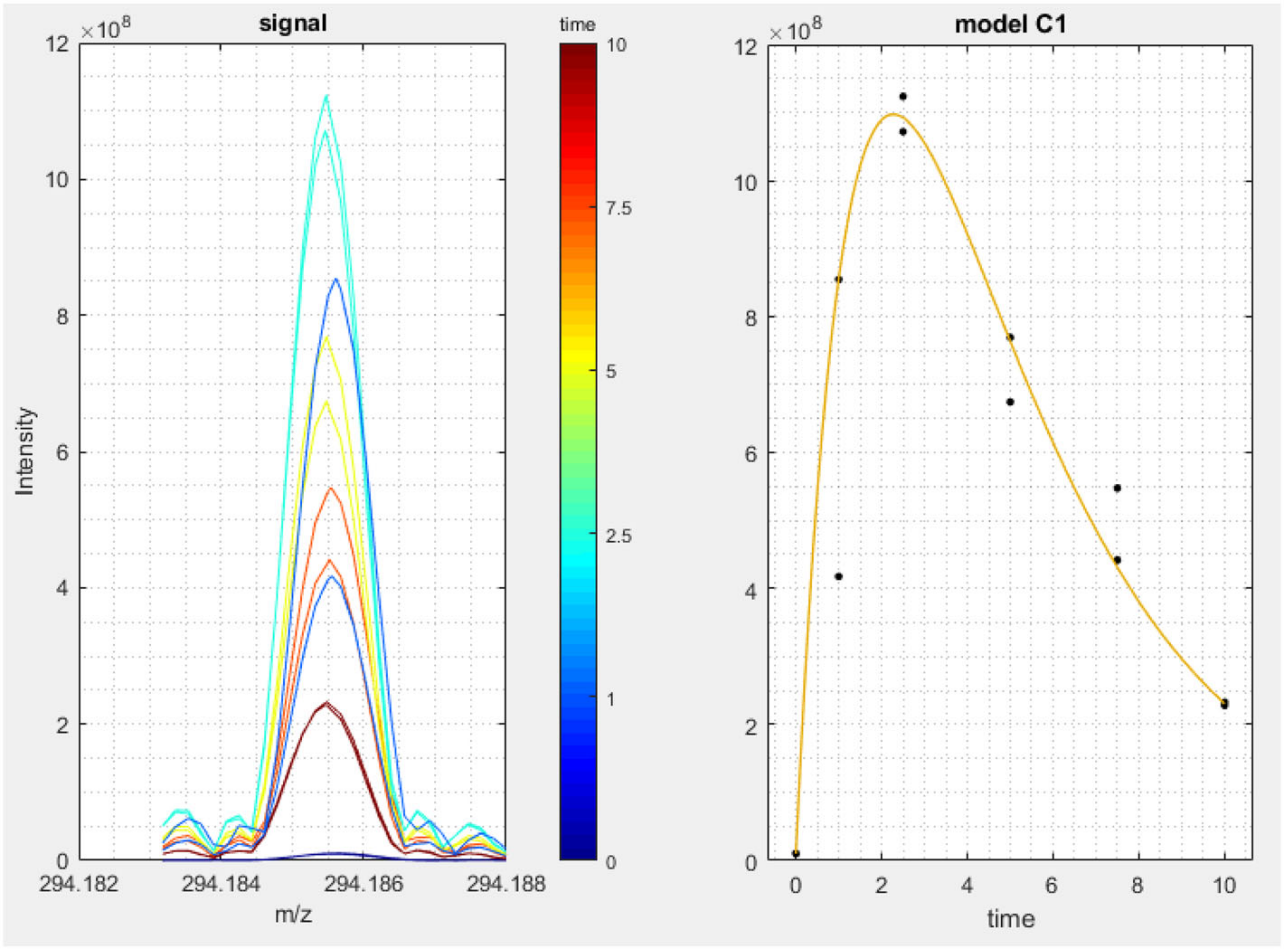
*m*/*z* 294.18552 signal extracted for each irradiation time and associated kinetic model

Using the selection parameters referred to above, one protonated species was detected at *m*/*z* 336.14927. Considering the “seven golden rules” of Kind and Fiehn and selecting atoms C, H, N, O, P, S, F, Cl, Br, Si, Na and K, the only matching formula was C_23_H_18_N_3_^+^.^28^ This species is logically assumed not to be related to maprotiline; it could correspond to a contaminant in high concentration in wastewater, degrading under UV radiation. According to the kinetics revealed by SPIX, some photoproducts were present in detectable abundance after 2.5 min irradiation. To estimate the relevance of the results provided by SPIX, one of the spectra recorded at this reaction time was selected. After blank subtraction (the blank consisting of wastewater matrix without maprotiline), the spectrum was exported in .csv format (Bruker's FTICR‐MS file format). The spectra were recorded using 8 Mpt, and as the experiments were carried out using secondary treated wastewater, 4479 peaks were exported by the Bruker software which were above the signal‐to‐noise ratio threshold of 4. Out of these 4479 peaks, the 11 most abundant ions in the selected spectrum corresponded to the 11 most statistically relevantly changing *m*/*z* values extracted by SPIX. The two *m*/*z* signals corresponding to harmonics of major ions were also extracted with good fit, and ranked 18th and 115th in the original file (overall blank‐subtracted spectrum). The photoproducts showing the highest significance (lowest *p* values) were those corresponding to *m*/*z* 294.18552 and *m*/*z* 302.17530 (protonated molecules); the fitting is presented in Figure 3 for the former. One of the maprotiline‐related peaks that was not in the list of the highest intensities was not found in the spectrum recorded at 2.5 min of irradiation: it was removed by the blank subtraction process, since the wastewater matrix was very complex. It is thus noteworthy that SPIX does not require blank subtraction to provide valuable information, allowing relevant peaks that coincidentally overlap with some of the matrix peaks not to be removed. The experiment conducted on photodegradation of maprotiline showed that the SPIX software efficiently revealed the most relevant changes in the composition of the irradiated mixture on the basis of only 12 mass spectra. It was able to automatically detect reagents, intermediates and products at trace levels. Most extracted ions were related either to maprotiline or to its photoproducts. Only one compound was found which was assumed not to be related to maprotiline on the basis of its molecular formula (C_23_H_17_N_3_); no significant change in the composition of the dissolved organic matter was found, although of course only electrospray‐ionized species were considered. The photodegradation pathways of maprotiline have been reported in a study more oriented toward structural elucidation.^29^

### 4.2 Comparison of two conditions: example of photodegradation of acetamiprid in aqueous solution of fulvic acid

**FIGURE 4:**
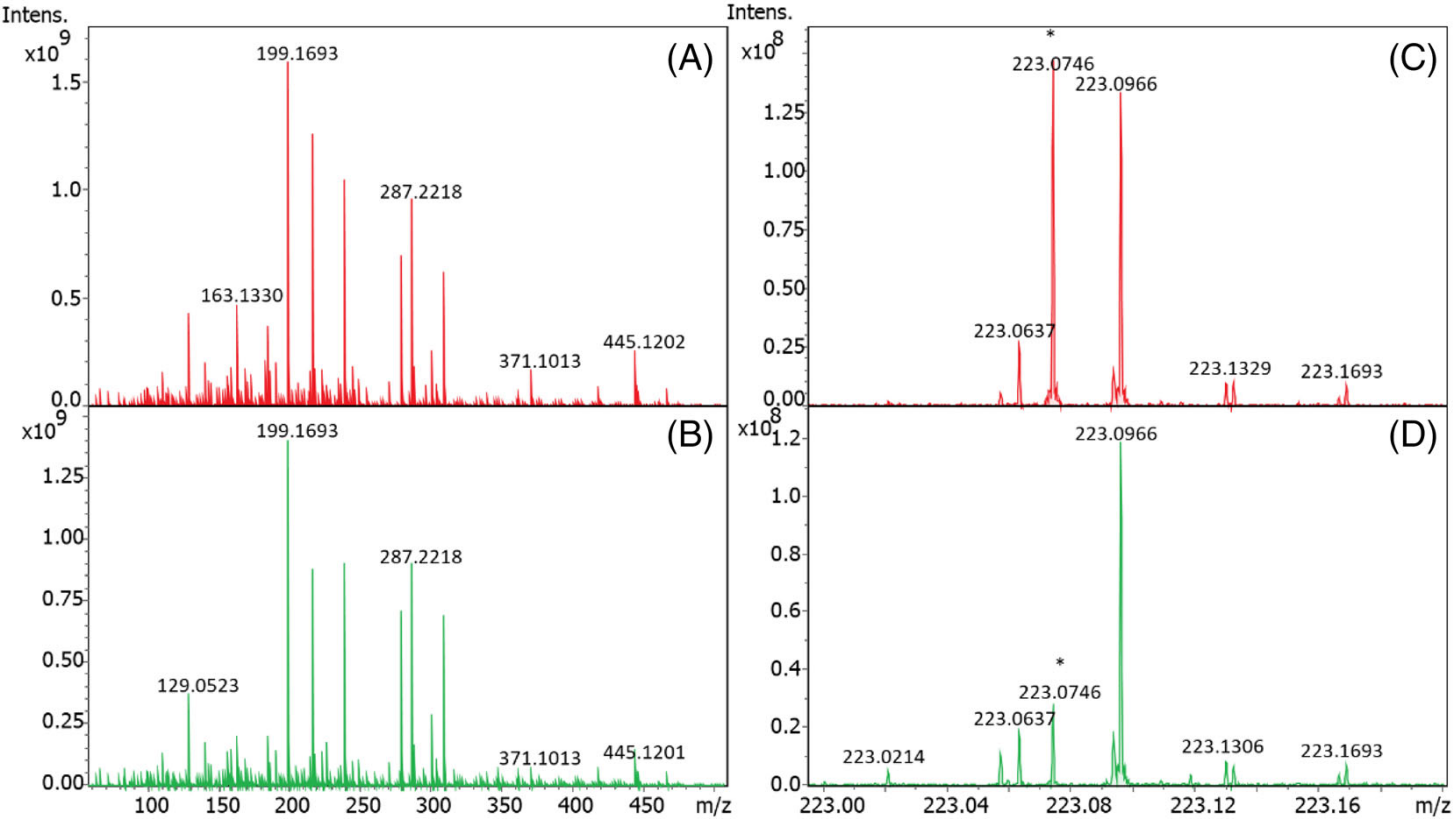
Mass spectra of acetamiprid in mixture with fulvic acid. A, Mass spectrum before photolysis; B, mass spectrum after 30 min photolysis; C, zoom on the protonated acetamiprid peak (*m*/*z* 223.0746) in (A); D, zoom on the protonated acetamiprid peak in (B)

This example demonstrates the ability of the SPIX software to point out relevant changes between two conditions from changes in low‐abundance ions within a complex matrix. We studied the effect of UV radiation on acetamiprid in a complex mixture, simulating river water. A prior photolysis study of acetamiprid in ultrapure water identified acetamiprid degradation products in ultrapure water and demonstrated that the presence of other substances in the matrix leads to the formation of different degradation products.^30^ These results led us to study the effect of dissolved organic matter on the photodegradation of acetamiprid as may happen under real environmental conditions. The peak intensity of acetamiprid represented 9.2% of the base peak of the mass spectrum before photolysis and only 1.9% after 30 min irradiation. A sodium adduct, originating from the use of glassware during sample preparation, impurity in solvents or electrospray ionization needle for instance, was detected with a relative intensity of 10.4% in the spectrum recorded before and 2.4% after photolysis. These differences, not detectable looking at the whole spectrum, are obvious when zooming on the region from *m*/*z* 223.00 to 223.20 (Figure 4).

**FIGURE 5:**
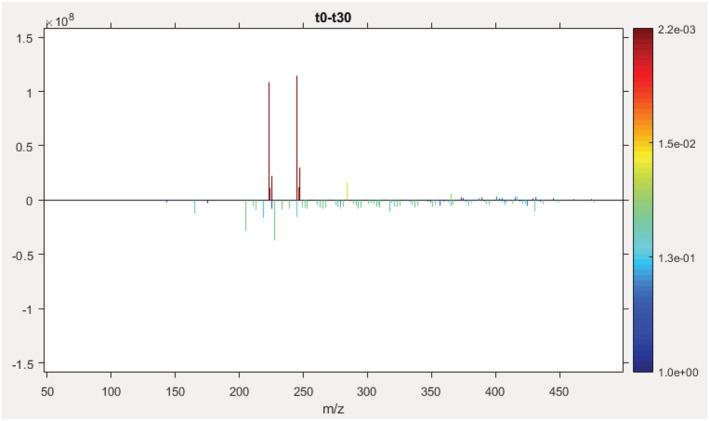
Visual result provided by the SPIX software after processing of mass spectra series recorded from samples taken before and after 30 min photolysis. The differences in ion intensities are given on the *y*‐axis, positive values corresponding to decreased intensity after irradiation. The associated *p* value is given by the color scale: the more the color tends toward red, the more statistically significant the difference

Sets of spectra recorded before and after photolysis were compared using the SPIX software. Given the intensity of acetamiprid within the mixture, a peak detection limit was set at only three times the average intensity of spectral noise (average noise at 1 × 10^6^, detection threshold set at 3 × 10^6^). This threshold was set as low as possible so as to identify acetamiprid degradation products in small amounts. Blank spectra were subtracted to eliminate any interference from solvents or instruments. Exported from SPIX, Table 2 lists the ions the abundance of which underwent significant change after irradiation. Here, only ions with a probability of ≥95% (*p*≤ 0.05) were retained, i.e. with significant difference in intensity between the two conditions. In this example, results are organized by increasing *p* value, but any parameter can be chosen for presentation of the results. A negative value in the “Difference” column indicates that the ion abundance increased after photolysis. Visual comparison (Figure 5) confirms the results displayed in Table 2: the greatest change in intensity – and with the highest significance – was the concomitant decrease of the *m*/*z* 223 (MH^+^) and *m*/*z* 245 (MNa^+^) ions. Many other peaks decreased or increased after photolysis, but with lower *p* values for the difference in intensity; some were related to acetamiprid photodegradation, others to changes in dissolved organic matter. The ions at *m*/*z* 205.10847 (C_10_H_13_N_4_O^+^) and *m*/*z* 227.09036 (C_10_H_12_N_4_NaO^+^) shown here correspond to the protonated and cationized forms of a photoproduct previously described, the structure of which was elucidated in a study of the UV irradiation of acetamiprid in pure water.^23^ This photoproduct was the major one. Thus, it is not possible to say whether others which were previously described were not detected here due to too high detection thresholds or because they are not formed in the presence of fulvic acid. It is interesting to note that SPIX revealed two ions, *m*/*z* 284.12725 (C_12_H_19_ClN_5_O^+^) and *m*/*z* 245.10095 (C_10_H_14_N_4_NaO_2_^+^) – resulting from ionization of acetamiprid photoproducts based on their formulae – that were not detected by LC/MS in pure water. With a relative intensity below 1% in infusion mode, these two molecules could not have been revealed without SPIX. Fifteen ions with abundance significantly varying before and after UV irradiation were indicated. Based on their chemical formulae, they were assumed not to be related to acetamiprid or to its photoproducts; they all included a large number of oxygen atoms (≥6) and probably resulted from oxidation of dissolved organic matter. This is of great interest because it opens a way to investigate the global consequences of a depollution treatment, evaluating the treatment – apart from its ability to efficiently degrade pollutants – in terms of biotope preservation.

**TABLE 2:** *m*/*z* values for which the intensity significantly varied between series of spectra recorded before and after 30 min of irradiation (*n* = 6). Ions are listed by increasing *p* value

*m*/*z*	Difference in intensity	*p* value	Ion formula	Related to acetamiprid^a^
224.07792	10 523 197	1.47 × 10^−6^	C_9_H_12_ClN_4_^^13^C^+^	Yes
223.07459	103 518 217	3.20 × 10^−6^	C_10_H_12_ClN_4_^+^	Yes
225.07161	24 820 606	1.08 × 10^−5^	C_10_H_12_N_4_^^37^Cl^+^	Yes
246.05988	10 966 361	1.30 × 10^−5^	C_9_H_11_ClN_4_Na^^13^C^+^	Yes
247.05358	30 754 403	2.86 × 10^−5^	C_10_H_11_N_4_Na^^37^Cl^+^	Yes
245.05653	107 329 585	4.50 × 10^−5^	C_10_H_11_ClN_4_Na^+^	Yes
201.03705	−3 542 730	1.84 × 10^−2^	C_6_H_10_NaO_6_^+^	No
205.10847	−28 497 851	2.11 × 10^−2^	C_10_H_13_N_4_O^+^	Yes
251.05269	−7 062 764	2.71 × 10^−2^	C_12_H_11_O_6_^+^	No
297.05819	−5 696 496	2.72 × 10^−2^	C_11_H_14_NaO_8_^+^	No
267.04760	−5 586 613	2.99 × 10^−2^	C_10_H_12_NaO_7_^+^	No
283.04253	−4 479 881	3.20 × 10^−2^	C_10_H_12_NaO_8_^+^	No
237.03704	−4 871 105	3.25 × 10^−2^	C_9_H_10_NaO_6_^+^	No
253.03194	−4 495 727	3.33 × 10^−2^	C_9_H_10_NaO_7_^+^	No
245.10095	−14 590 229	3.65 × 10^−2^	C_10_H_14_N_4_NaO_2_^+^	Yes
211.02138	−5 849 796	3.65 × 10^−2^	C_7_H_8_NaO_6_^+^	No
227.09036	−38 558 966	3.70 × 10^−2^	C_10_H_12_N_4_NaO^+^	Yes
239.05270	−7 601 354	3.77 × 10^−2^	C_9_H_12_NaO_6_^+^	No
213.03705	−8 698 910	4.00 × 10^−2^	C_7_H_10_NaO_6_^+^	No
275.05267	−3 822 758	4.02 × 10^−2^	C_12_H_12_NaO_6_^+^	No
284.12725	12 781 460	4.41 × 10^−2^	C_12_H_19_ClN_5_O^+^	Yes
253.06834	−7 132 886	4.57 × 10^−2^	C_10_H_14_NaO_6_^+^	No
255.08400	−3 840 684	4.60 × 10^−2^	C_10_H_16_NaO_6_^+^	No
325.05308	−3 163 824	4.66 × 10^−2^	C_12_H_14_NaO_9_^+^	No
241.03195	−4 410 055	4.98 × 10^−2^	C_8_H_10_NaO_7_^+^	No

^a^ Assumption based on the ion chemical formula.

## 5 CONCLUSIONS

The SPIX software aims at extracting relevant data from mass spectra datasets. User‐friendly and totally free, it is available for all at: http://spix.webpopix.org. Two features of SPIX are presented in this article, based on examples taken from the field of environmental analysis. The first example showed how SPIX revealed photodegradation reactions by correlating significant changes in ion abundance over time with kinetic models. Thus, the software revealed the reagents, products and intermediate species in a very complex mixture (wastewater). This functionality can be extended to monitor any kind of reaction – even unknown – in all types of mixtures. Some features of the SPIX software are still under development. One aims at extending compatibility with many more file formats (wiff, .d, .pkl, .qgd, etc.), in order to be used with most marketed mass spectrometers. Another focus of development – already running but requiring some improvements – concerns the extension of SPIX to three‐dimensional datasets from hyphenated techniques such as GC/MS, LC/MS or IM‐MS. Some commercial software applications allow comparison of chromatograms but, to our knowledge, an approach consisting of extracting relevant data from hyphenated techniques based on fitting with kinetic models has never been reported. The second example showed the ability of SPIX to detect photoproducts at trace amounts in an aqueous solution containing dissolved organic matter, through comparison of datasets for two conditions (before/after photolysis in the present case). Regarding this example, the ability of SPIX to deal with intrinsic variability and reduce operator subjectivity opens up promising prospects in all areas of analytical chemistry. It could, in particular, be a very useful tool to assess fragrance and flavor counterfeits, where assessment is very challenging due to the normal variations in abundance of natural substances featuring at low levels in their composition. This feature can also be used to estimate the global consequences of a given treatment on the treated medium, for example to monitor the oxidation of dissolved organic matter and its consequences on biotope preservation.

6

### PEER REVIEW

The peer review history for this article is available at https://publons.com/publon/10.1002/rcm.9015.

## Supporting information


**Data SI‐1.** State of the art of modern approaches in managing high‐resolution mass spectrometry data
SI‐2. Current kinetic models in SPIX
SI‐3. Chemicals, reagents and sample preparation
SI‐4. Exported data from the SPIX software after assignation of a kinetic model
