# Supplementary material for: SPIX: A new software package to reveal chemical reactions at trace amounts in very complex mixtures from high‐resolution mass spectra dataset
Source: Rapid Commun Mass Spectrom. 2021 Jan 20;35(6):e9015. doi: 10.1002/rcm.9015 (PMC7900974; doi:10.1002/rcm.9015)
Supplement: Supplementary file 1 — Data SI‐1. State of the art of modern approaches in managing high‐resolution mass spectrometry data SI‐2. Current kinetic models in SPIX SI‐3. Chemicals, reagents and sample preparation SI‐4. Exported data from the SPIX software after assignation of a kinetic model [file RCM-35-e9015-s001.docx]

**Supporting information**

SPIX: a new software package to reveal chemical reactions at trace amounts in very complex mixtures from high-resolution mass spectra data sets

Edith Nicol, Yao Xu, Zsuzsanna Varga, Said Kinani, Stéphane Bouchonnet, Marc Lavielle

**SI-1**. State of the art of modern approaches in managing high-resolution mass spectrometry data

**SI-2**. Current kinetic models in SPIX

**SI-3**. Chemicals, reagents and sample preparation

**SI-4**. Exported data from the SPIX software after assignation of a kinetic model

**SI-1. State of the art of modern approaches in managing high-resolution mass spectrometry data**

In mass spectrometry, the emergence of high-resolution analyzers has enabled analysis of samples of ever-increasing complexity. Whether in direct infusion or with hyphenated techniques (LC/MS and GC/MS couplings), the amount of information issuing from high-resolution analysis of complex mixtures requires computer processing and simplified data representation. Direct infusion of a sample can thus provide a mass spectrum including several thousands of distinct ions. Various representations are commonly used to simplify the visualization and comparison of samples analyzed by mass spectrometry. As the approaches are so diverse, this section does not seek to be exhaustive, but restricts itself to presenting the Kendrick and Van Krevelen diagrams and the multivariate statistical analyses most commonly used by high-resolution mass spectrometry specialists.

***Kendrick diagrams***

The Kendrick diagram allows easy identification, from a mass spectrum, of series of compounds that include the same number of heteroatoms and unsaturations but differ from each other by the number of -CH_2_- groups.^[[1]](#footnote-1)^ The diagram is built by plotting the Kendrick mass defect (KMD) for each ion (eq. 1) as a function of the Kendrick mass (KM) (eq. 2).

$KMD=\left( Nominal Kendrick Mass-Exact Kendrick Mass \right)$ (eq. 1)

$KM=IUPAC mass*\left( \frac{14}{14.01565} \right)$ (2)

Compounds in the same series (i.e., with the same number of heteroatoms and degrees of unsaturation) will have the same KMD. In the diagram, each series is aligned horizontally with a deviation of 14 that reflects a difference of one -CH_2_- pattern. A shift of 0.01340 on the vertical axis corresponds to implementation of 1 unsaturation. Originally reported for the investigation of petroleomics-type samples in the early 2000s,^[[2]](#footnote-2),^^[[3]](#footnote-3)^ the use of the Kendrick diagram has been extended and adapted over the years for complex environmental samples,^[[4]](#footnote-4),^^[[5]](#footnote-5),^^[[6]](#footnote-6)^ metabolomic studies,^[[7]](#footnote-7),^^[[8]](#footnote-8)^ and proteomics on phosphopeptides.^[[9]](#footnote-9)^ As needs differ between environmental chemistry and petroleomics, many studies have focused on modification of the mass defect^[[10]](#footnote-10)^ in order to characterize reaction products such as oxidation or chlorination.^[[11]](#footnote-11),^^[[12]](#footnote-12)^ Even with a mass measurement accuracy of 1 ppm, a compound with a mass of 200 Da is assigned only 1 raw formula, while one with a mass of 500 Da is assigned 21.^[[13]](#footnote-13)^ Regarding this issue, the Kendrick diagram can significantly increase the number of single raw formulae that can be assigned from *m/z* values in a mass spectrum. From the raw formula of the first compound, the identification of homologous series aids in assigning the raw formulae of other compounds in the series regardless of their mass. This allows a complex spectrum to be recalibrated, to obtain the best possible accuracy and thus assign as many raw formulae as possible (e.g., prior to a principal component analysis).^[[14]](#footnote-14),^^[[15]](#footnote-15)^

***Van Krevelen diagrams***

The van Krevelen diagram, originally used in petroleomics to control oil and kerosene quality, represents the H/C ratio as a function of the O/C or N/C ratio for each ion of a complex mixture.^[[16]](#footnote-16)^ This allows the composition of a sample to be quickly estimated based on constituent molecular families (lipids, proteins, sugars, carbohydrates, lignin, tannins, etc.).^[[17]](#footnote-17)^ Today, this representation is commonly used for environmental samples to track their evolution following an event such as treatment or pollution.^[[18]](#footnote-18),^^[[19]](#footnote-19),^^[[20]](#footnote-20),^^[[21]](#footnote-21),^^[[22]](#footnote-22)^ Some studies have extended the van Krevelen diagram over 3 dimensions to achieve better classification of compounds and better differentiation between complex mixtures. In some cases, this approach is associated with other t-test type statistical tests.^[[23]](#footnote-23),^^[[24]](#footnote-24)^

***Multivariate statistical analysis***

Multivariate statistical analysis is a versatile tool for dealing with high-dimensional datasets, and many methods can be used to extract valuable information, perform data compression, assess subclasses and compare groups of samples assessing relationships between variables. For quantitative datasets, two categories of model can be distinguished in terms of the relationship between variables and response, according to the parameters: linear and non-linear. An example is UV-Vis absorbance analysis of a complex mixture, where the absorbance depends on the concentrations of all the compounds present in the mixture, based on a linear relationship; in this case, multivariate linear regression can describe the correlations.^[[25]](#footnote-25)^ On the other hand, supervised and unsupervised methods are applied for qualitative datasets.^[[26]](#footnote-26),^^[[27]](#footnote-27)^ Principal component analysis, an unsupervised method, is usually implemented as a first approach for visualization, dimensionality reduction, classification, and finding patterns of similarities in the dataset.^[[28]](#footnote-28),^^[[29]](#footnote-29)^ Supervised methods require *a priori* information, meaning that classes, determined by specific qualitative properties, are known in advance, and this information is used to sharpen the distinction between the given classes. A subclass of these methods, discriminant analysis, studies why the classes are different and which variables drive their separation, bearing the largest discriminatory power (e.g., Partial Least Squares Discriminant Analysis).^[[30]](#footnote-30)^ The main areas of application in mass spectrometry data interpretation include food analysis and authentication,^[[31]](#footnote-31),^^[[32]](#footnote-32),^^[[33]](#footnote-33),^^[[34]](#footnote-34)^ environmental sample analysis,^[[35]](#footnote-35),^^[[36]](#footnote-36)^ proteomics,^[[37]](#footnote-37),^^[[38]](#footnote-38)^ metabolomics in diagnostics and biology,^[[39]](#footnote-39),^^[[40]](#footnote-40),^^[[41]](#footnote-41)^ and imaging.^[[42]](#footnote-42),^^[[43]](#footnote-43)^ However, when dealing with high-dimensionality data (e.g., direct infusion HRMS) it is difficult to assess and visualize which variables account for the differences. Usually, variable selection^[[44]](#footnote-44)^ or sparse methods^[[45]](#footnote-45)^ must be applied, which are able to remove or suppress variables that are irrelevant to response prediction or classification.^[[46]](#footnote-46)^ These methods proved their efficiency but have to be used with caution to avoid losing valuable information, to prevent overfitting, and to handle chance correlations correctly. In summary, multivariate analysis tools enable global understanding of many concomitant variables and of their inter-correlations. The concept behind multivariate analysis is different from that of the SPIX software: the latter aims at observing all statistically relevant variables individually.

**SI-2. Current kinetic models in SPIX**


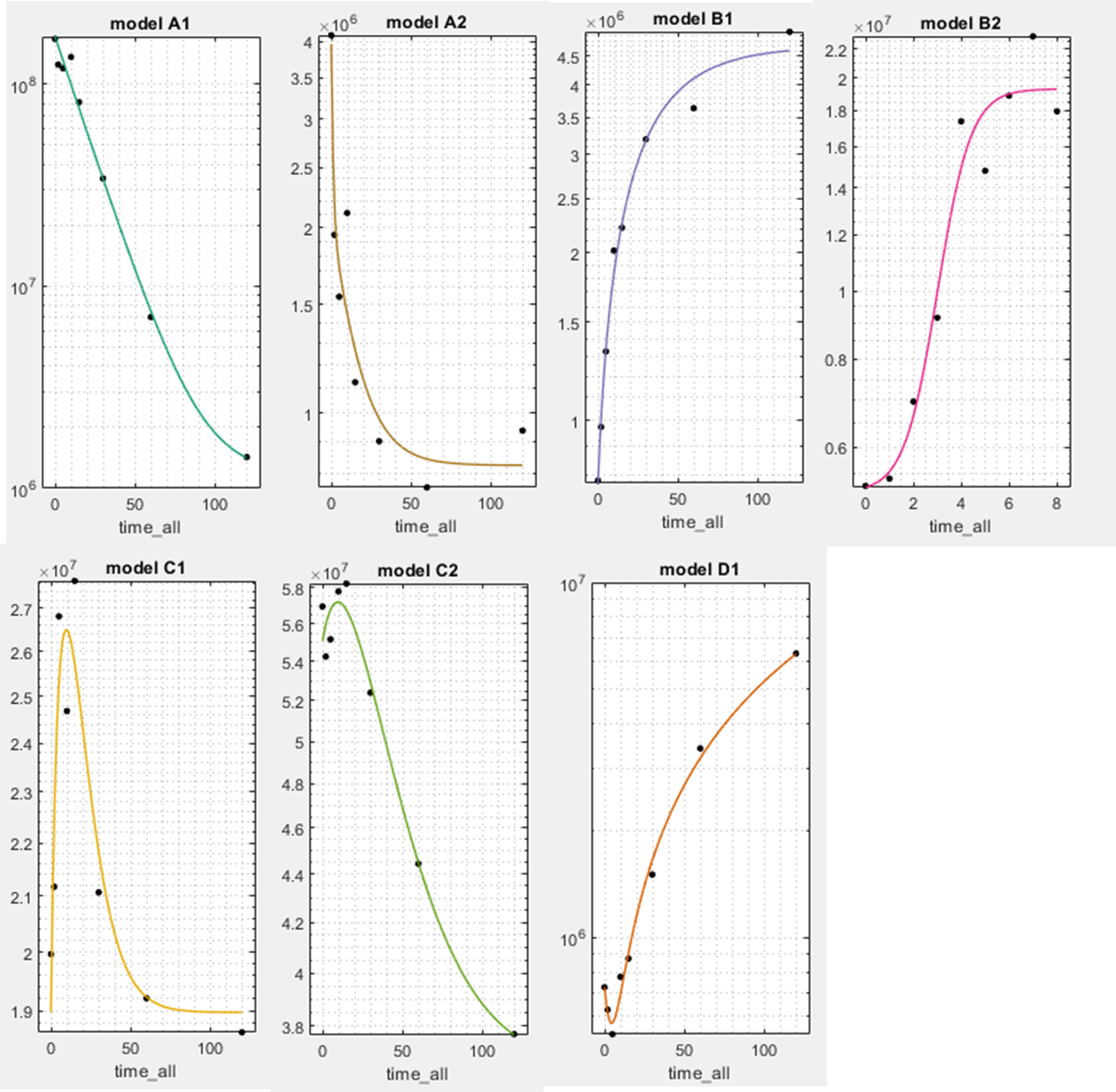


| Model | Theoretical equation | Experimental equations for the examples shown |
| --- | --- | --- |
| A1 | f=f0+A*exp(-k*t)  k>0 | 1.18E+06 + 1.70E+08 * exp(-5.50E-02 * t) |
| A2 | f=f0+A1*exp(-k1*t)+A2*exp(-k2*t)  k1>0 ; k2>0 | 8.22E+05 + 1.89E+06 * exp(-9.61E-01 * t) + 1.25E+06 * exp(-6.93E-02 * t) |
| B1 | f=f0+B*(1-exp(-k*t))  k>0 | 4.69E+06 - 3.91E+06 * exp(-3.17E-02 * t) |
| B2 | f=A+B/(1+C*exp(-k*t))  k>0 | 1.21E-12 + 1.47E+07 / (1 + exp(-4.83E-02 * (t - 65.23))) |
| C1 | f=f0+A*(exp(-k1*t)-exp(-k2*t))  k2 > k1 > 0 | 1.90E+07 + 3.25E+08 * (exp(-1.00E-01 * t) - exp(-1.06E-01 * t)) |
| C2 | f=f0+A1*exp(-k1*t)-A2*exp(-k2*t)  k2 > k1 > 0 ; A1 > A2 | 3.64E+07 + 3.70E+08 * exp(-3.96E-02 * t) - 3.52E+08 * exp(-4.29E-02 * t) |
| D1 | f=f0+A1*exp(-k1*t)-A2*exp(-k2*t)  k1 > k2 > 0 ; A2 > A1 | 1.01E+09 + 6.22E+05 * exp(-2.14E-01 * t) - 1.01E+09 * exp(-5.12E-05 * t) |

**SI-3. Chemicals, reagents, irradiation processes and sample preparation**

**Chemicals and reagents**

Acetamiprid ((E)-N-(6-chloro-3-pyridylmethyl)-N’-cyano-N-methylacetamidine), maprotiline hydrochloride (*N*-methyl-3-(1-tetracyclo[6.6.2.0^2,7^.0^9,14^]hexadeca-2,4,6,9,11,13-hexaenyl) propan-1-amine;hydrochloride), acetonitrile (ACN) and formic acid (FA) (chromatographic grade purity > 99.99% for both) were purchased from Sigma-Aldrich (Saint Quentin Fallavier, France). Suwannee River Fulvic Acid Standard was purchased from International Humic Substances Society (Denver, CO, USA). Ultrapure water (specific resistance, 18 MΩ cm^-1^ at 25 °C) was produced by a Purelab Chorus 1 water purification system purchased from Veolia Water Technologies (Wissous, France).

**Sample preparation**

***1. Peroxide/UV photodegradation of maprotiline in wastewater***

The UV photocatalyzed degradation of maprotiline was carried out in a 45-L pilot plant with continuous flow at a wastewater treatment plant operated by the FACSA company in Alhama de Murcia, Spain. The molecule was submitted to peroxide/UV advanced oxidation process. The pilot plant included a tank for mixing with a stirrer, a reactor with UV lamp (model UVLA-325-4, controlled by a Synergy 3 control panel - ATG UV Technology, Wigan, UK), a pump for water circulation, a rotameter to assess the water flow, and a compressor (Metabo Basic 250-50 W, Metabowerke, Nürtingen, Germany) to provide airflow in the rotameter equipped-reactor. The wastewater, secondary treated water originating from municipal and industrial sources, was transferred into the pilot plant after undergoing preliminary treatment (screening, sand and grease removal): decantation, biological treatment and sand filtration, before the chlorination step. At this point there were still bacteria in the mixture as well as micropollutants, which the traditional wastewater treatment methods are not able to remove. The wastewater was spiked with Maprotiline hydrochloride at 5 ppm, and the total organic carbon content of the mixture was measured as 37.2 mg/L. 4 mL of hydrogen peroxide of technical grade (33 v/w%, VWR chemicals, Llinars del Vallès, Spain) were added to the pilot plant; this corresponds to the stoichiometric amount needed for Maprotiline mineralization. Since the reaction with peroxide radical is usually fast, the reaction time was 10 minutes and the sampling for HRMS (2 x 1 mL) was done at the following times: t0 (3 samples), 1 min, 2.5 min, 5 min, 7.5 min and 10 min. The only sample preparation before direct infusion mass spectrometry analysis was the addition of 0.1 mL of acetonitrile and 0.1% of formic acid to the 1-mL samples, to achieve better ionization and solubility. Direct infusion HRMS was aimed at suppressing many sample preparation steps, to gain a considerable amount of time and avoid too much variability and operator subjectivity.

***2. UV irradiation of acetamiprid in an aqueous solution of humic acid***

Acetamiprid has been detected in agriculture water samples at concentrations of up to 44 µg/L.^[[47]](#footnote-47)^ Therefore, a 40 µg/L acetamiprid solution was prepared using an aqueous solution of fulvic acid at 20 mg/L, a mean value corresponding to the amount usually found in natural waters.^[[48]](#footnote-48)^ Six glass tubes of the solution were simultaneously irradiated for 30 minutes in a laboratory-made reactor equipped with a UV-Vis high-pressure mercury lamp HPL-N125W/542 E27 SC (Philips, Ivry-sur-Seine, France) emitting light at wavelengths ranging from 200 nm to 650 nm, with a maximum irradiation wavelength at 254 nm and a radiation flux of 6200 lm. Each tube contained 50 mL of solution to ensure good surface irradiation. 1 mL of solution was taken twice from each tube before and after irradiation. Samples were analyzed by electrospray ionization-MS using automated direct infusion with a solvent made up of 50% H_2_O/AF (0.1%) and 50% ACN/AF (0.1%) at a flow rate of 0.002 mL/min. The six replicates and blanks (H_2_O/ACN 50/50 v/v) were randomly analyzed and data were extracted in a «.xy » text format so that they could be treated with the SPIX software.

**SI-4. Exported data from the SPIX software after assignation of a kinetic model to a *m/z* ratio**

| Segment | *m/z* | | Intensity | Time | | File |
| --- | --- | --- | --- | --- | --- | --- |
| 65 | 278.19055 | | 3.53E+10 | 0.0 | | Maprotiline WW H2O2 0min B.xy |
| 65 | 278.19057 | | 3.39E+10 | 1.0 | | Maprotiline WW H2O2 1min B.xy |
| 65 | 278.19050 | | 8,34E+09 | 2.5 | | Maprotiline WW H2O2 2min B.xy |
| 65 | 278.19052 | | 3,74E+09 | 5.0 | | Maprotiline WW H2O2 5min B.xy |
| 65 | 278.19057 | | 1,95E+09 | 7.5 | | Maprotiline WW H2O2 7min B.xy |
| 65 | 278.19053 | | 8,84E+08 | 10.0 | | Maprotiline WW H2O2 10min B.xy |
|  | | | | |  |  |
| m/z | r^2^ | p-value | | model |  |  |
| 278.19053 | 0.99 | 0.000776 | | A1 |  |  |

1. Kendrick EA mass scale based on CH_2_ = 14.0000 for high resolution mass spectrometry of organic compounds. *Anal Chem.* **1963**; *35(13)*” 2146-2154 [↑](#footnote-ref-1)
2. Marshal G, Rodgers RP. Petroleomics: the next grand challenge for chemical analysis. *Acc Chem Res.* **2004**, *37*, 53-59 [↑](#footnote-ref-2)
3. Hughey A, Hendrickson CL, Rodgers RP, Marshall AG. Kendrick mass defect spectrum: a compact visual analysis for ultrahigh-resolution broadband mass spectra. *Anal Chem.* **2001**, *73*, 4676-4681 [↑](#footnote-ref-3)
4. Chu FL, Pirastru, L.; Popovic, R.; Sleno, L. Carotenogenesis up-regulation in Scenedesmus sp. using a targeted metabolomics approach by liquid chromatography – high-resolution mass spectrometry. *J. Agric. Food Chem*. **2011**, *59*, 3004-3013 [↑](#footnote-ref-4)
5. Sleighter, R.L.; Hatcher, P.G. The application of electrospray ionization coupled to ultrahigh resolution mass spectrometry for the molecular characterization of natural organic matter. *J. Mass Spectrom*. **2007**, *42*, 559-574 [↑](#footnote-ref-5)
6. Kramer, R.W.; Kujawinski, E.B.; Hatcher, P.G. Identification of black carbon derived structures in a volcanic ash soil humic acid by Fourier transform ion cyclotron resonance mass spectrometry. *Environ. Sci. Technol*. **2004**, *38*, 3387-3395 [↑](#footnote-ref-6)
7. Ni, S.; Qian, D.; Duan, J, et al. UPLC-QTOF/MS-based screening and identification of the constituents and their metabolites in rat plasma and urine after oral administration of Glechoma longituba extract. *J. Chromatogr. B* **2010**, *878*, 2741-2750 [↑](#footnote-ref-7)
8. Zhang, H.; Zhang, D.; Ray, K.; Zhu, M. Mass defect filter technique and its applications to drug metabolite identification by high-resolution mass spectrometry. *J. Mass Spectrom*. **2009**, *44*, 999-1016 [↑](#footnote-ref-8)
9. Bruce, C.; Shifman, M.A.; Miller, P.; Gulcicek, E.E. Probabilistic enrichment of phosphopeptides by their mass defect. *Anal. Chem*. **2006**, *78*, 4374-4382 [↑](#footnote-ref-9)
10. Sleno, L. The use of mass defect in modern mass spectrometry. *J. Mass. Spectrom*. **2012**, *47*, 226-236 [↑](#footnote-ref-10)
11. Jobst, K.J.; Shen, L.; Reiner, E.J, et al. The use of mass defect plots for the identification of (novel)halogenated contaminants in the environment, *Anal. Bioanal. Chem*. **2013**, *405*, 3289-3297 [↑](#footnote-ref-11)
12. Taguchi, V.Y.; Nieckarz, R.J.; Clement, R.E.; Krolik, S.; Williams, R. Dioxin analysis by gas chromatography-Fourier transform ion cyclotron resonance mass spectrometry (GC-FTICRMS). *J. Am. Soc. Mass Spectrom.* **2010**, *21*, 1918-1921 [↑](#footnote-ref-12)
13. Kind, T.; Fiehn, O. Metabolomic database annotations via query of elemental compositions: Mass accuracy is insufficient even at less than 1 ppm. *BMC Bioinform.* **2006**, 7, 234-243 [↑](#footnote-ref-13)
14. Ajaero, C.; McMartin, D.W.; Peru, K.M, et al. Fourier transform ion cyclotron resonance mass spectrometry characterization of Athabasca oil sand process-affected waters incubated in the presence of wetland plants. *Energy Fuels* **2017**, *31*, 1731-1740 [↑](#footnote-ref-14)
15. Ajaero, C.; Peru, K.M.; Hughes, S.A, et al. Atmospheric pressure photoionization fourier transform ion cyclotron resonance mass spectrometry characterization of oil sand process-affected water in constructed wetland treatment. *Energy Fuels* **2019**, *33*, 4420-4431 [↑](#footnote-ref-15)
16. van Krevelen, D.W. Graphical-statistical method for the study of structure and reaction processes of coal. *Fuel* **1950**, *29*, 269-284 [↑](#footnote-ref-16)
17. Kew, W.; Blackburn, J.W.T.; Clarke, D.J.; Uhrín, D. Interactive van Krevelen diagrams – advanced visualisation of mass spectrometry data of complex mixtures. *Rapid Commun. Mass Spectrom.* **2017**, *31*, 658-662 [↑](#footnote-ref-17)
18. Minor, E.C.; Swenson, M.M.; Mattson, B.M.; Oyler, A.R. Structural characterization of dissolved organic matter: a review of current techniques for isolation and analysis. *Environ. Sci.-Proc. Imp.* **2014**, *16*, 2064-2079 [↑](#footnote-ref-18)
19. D’Andrilli, J.; Foreman, C.M.; Marshall, A.G.; McKnight, D.M. Characterization of IHSS Pony Lake fulvic acid dissolved organic matter by electrospray ionization Fourier transform ion cyclotron resonance mass spectrometry and fluorescence spectroscopy. *Org. Geochem*. **2013**, *65*, 19-28 [↑](#footnote-ref-19)
20. Wozniak, A.S.; Bauer, J.E.; Sleighter, R.L.; Dickhut, R.M.; Hatcher, P.G. Technical Note: Molecular characterization of aerosol-derived water-soluble organic carbon using ultrahigh resolution electrospray ionization Fourier transform ion cyclotron resonance mass spectrometry. *Atmos. Chem. Phys.* **2008**, *8*, 5099-5111 [↑](#footnote-ref-20)
21. Hertkorn, N.; Benner, R.; Frommberger, M, et al. Characterization of a major refractory component of marine dissolved organic matter. *Geochim. Cosmochim. Acta* **2006**, *70*, 2990-3010 [↑](#footnote-ref-21)
22. Kim, S.; Kramer, R.W.; Hatcher, P.G. Graphical method for analysis of ultrahigh-resolution broadband mass spectra of natural organic matter, the van Krevelen diagram. *Anal. Chem*. **2003**, *75*, 5336-5344 [↑](#footnote-ref-22)
23. Martins, N.; Jiménez-Morillo, N.T.; Freitas, F.; Garcia, R.; Gomez da Silva, M.; Cabrita, M.J. Revisiting 3D van Krevelen diagrams as a tool for the visualization of volatile profile of varietal olive oils from Alentejo region, Portugal. *Talanta* **2020**, *207*, 120276-120285 [↑](#footnote-ref-23)
24. Wu, Z.; Rodgers, R.P.; Marshall, A.G. Two- and three-dimensional van Krevelen Diagrams: A graphical analysis complementary to the Kendrick mass plot for sorting elemental compositions of complex organic mixtures based on ultrahigh-resolution broadband Fourier Transform ion cyclotron resonance mass measurements. *Anal. Chem.* **2004**, *76*, 2511-2516 [↑](#footnote-ref-24)
25. Massart, D.L.; Vandeginste, B.G.M.; Deming, S.M.; Michotte, Y.; Kaufman, L. Chemometrics: a textbook. **1988**, 165-182 [↑](#footnote-ref-25)
26. Jurs, P.C. Pattern recognition used to investigate multivariate data in analytical chemistry. *Science*, **1986**, *232*, 1219-1224 [↑](#footnote-ref-26)
27. Kemsley, E.K. Discriminant analysis of high-dimensional data: a comparison of principal components analysis and partial least squares data reduction methods. *Chemom. Intell. Lab. Syst.* **1996**, *33*, 47-61 [↑](#footnote-ref-27)
28. Abdi, H.; Williams, L.J. Principal component analysis. *WIREs Comput. Stat.* **2010**, *2*, 433-459 [↑](#footnote-ref-28)
29. Ringnér, M. What is principal component analysis?, *Nat. Biotechnol*. **2008**, *26(3),* 303-304 [↑](#footnote-ref-29)
30. Bylesjö, M.; Rantalainen, M.; Cloarec, O.; Nicholson, J.K.; Holmes, E.; Trygg, J. OPLS discriminant analysis: combining the strengths of PLS-DA and SIMCA classification. *J. Chemom*. **2006**, *20*, 341-351 [↑](#footnote-ref-30)
31. Callao, M.P.; Ruisanchez, I. An overview of multivariate qualitative methods for food fraud detection. *Food Control* **2018**, *86*, 283-293 [↑](#footnote-ref-31)
32. Marti, M.P.; Busto, O.; Guasch, J. Application of a headspace mass spectrometry system to the differentiation and classification of wines according to their origin, variety and ageing. *J. Chromatogr. A* **2004**, *1057*, 211-217 [↑](#footnote-ref-32)
33. Kenar, A.; Çiçek, B.; Arslan, F.N.; Akin, G.; Karuk Elmas, S.N.; Yilmaz, I. Electron impact-mass spectrometry fingerprinting and chemometrics for rapid assessment of authenticity of edible oils based on fatty acid profiling. *Food Anal. Methods* **2019**, *12*, 1369-1381 [↑](#footnote-ref-33)
34. Rubert, J.; Lacina, O.; Zachariasova, M.; Hajslova, J. Saffron authentication based on liquid chromatography high resolution tandem mass spectrometry and multivariate data analysis. *Food Chem.* **2016**, *204*, 201-209 [↑](#footnote-ref-34)
35. Karpuzcu, M.E.; Fairbairn, D.; Arnold, W.A, et al. Identifying sources of emerging organic principal components analysis. *Environ. Sci. Process. Impacts* **2014**, *16*, 2390-2399 [↑](#footnote-ref-35)
36. Corilo, Y.E.; Podgorski, D.C.; McKenna, A.M, et al. Oil spill source identification by principal component analysis of electrospray ionization fourier transform ion cyclotron resonance mass spectra. *Anal. Chem*. **2013**, *85*, 9064-9069 [↑](#footnote-ref-36)
37. Gaspari, M.; Verhoeckx, K.C.M.; Verheij, E.R.; van der Greef, J. Integration of two-dimensional lc-ms with multivariate statistics for comparative analysis of proteomic samples. *Anal. Chem.* **2006**, *78*, 2286-2296 [↑](#footnote-ref-37)
38. Wang, X.; Chambers, M.C.; Vega-montoto, J.; Bunk, D.M.; Stein, S.E.; Tabb, D.L. QC metrics from CPTAC Raw LC-MS/MS data interpreted through multivariate statistics. *Anal. Chem*. **2014**, *86*, 2497-2509 [↑](#footnote-ref-38)
39. Wang, C.; Kong, H.; Guan, Y, et al. Plasma phospholipid metabolic profiling and biomarkers of type 2 diabetes mellitus based on high-performance liquid chromatography/electrospray mass spectrometry and multivariate statistical analysis. *Anal. Chem*. **2005**, *77*, 4108-4116 [↑](#footnote-ref-39)
40. A. Kiss, A.; Lucio, M.; Fildier, A.; Buisson, C.; Schmitt-Kopplin, P.; Cren-Olivé, C. Doping control using high and ultra-high resolution mass spectrometry based non-targeted metabolomics - a case study of salbutamol and budesonide abuse. *PLoS One* **2013**, *8*, 1-13 [↑](#footnote-ref-40)
41. Tsugawa, H.; Tsujimoto, Y.; Arita, M.; Bamba, T.; Fukusaki, E. GC/MS based metabolomics: development of a data mining system for metabolite identification by using soft independent modeling of class analogy (SIMCA). *BMC Bioinform*. **2011**, *12*, 131-144 [↑](#footnote-ref-41)
42. Dill, A.L.; [Eberlin](https://www.ncbi.nlm.nih.gov/pubmed/?term=Eberlin%20LS%5BAuthor%5D&cauthor=true&cauthor_uid=20953777), L.S.; [Zheng](https://www.ncbi.nlm.nih.gov/pubmed/?term=Zheng%20C%5BAuthor%5D&cauthor=true&cauthor_uid=20953777), C, et al. Multivariate statistical differentiation of renal cell carcinomas based on lipidomic analysis by ambient ionization imaging mass spectrometry. *Anal. Bioanal. Chem*. **2010**, *398*, 2969-2978 [↑](#footnote-ref-42)
43. Alexandrov, T. MALDI imaging mass spectrometry: statistical data analysis and current computational challenges. *BMC Bioinform*. **2012**, *13*, S16-S11 [↑](#footnote-ref-43)
44. Peres, F.A.P.; Fogliatto, F.S. Variable selection methods in multivariate statistical process control: A systematic literature review. *Comput. Ind. Eng.* **2018**, *115*, 603-619 [↑](#footnote-ref-44)
45. Zou, H.; Hastie, T.; Tibshirani, R. Sparse principal component analysis. *J. Comput. Graph. Stat*. **2006**, *15,* 265-286 [↑](#footnote-ref-45)
46. Filzmoser, P.; Gschwandtner, M.; Todorov, V. Review of sparse methods in regression and classification with application to chemometrics. *J. Chemom*. **2012**, *26*, 42-51 [↑](#footnote-ref-46)
47. Anderson, T.A.; Salice, C.J.; Erickson, R.A.; McMurry, S.T.; Cox, S.B.; Smith, L.M. Effects of landuse and precipitation on pesticides and water quality in playa lakes of the southern high plains. *Chemosphere*, **2013**, *92, issue 1*, 84-90 [↑](#footnote-ref-47)
48. Thurman, E.M. Amount of organic carbon in natural waters. In: Organic geochemistry of natural waters, *Springer, Dordrecht*, **1985***, 2*, 7–65 [↑](#footnote-ref-48)
